# Identifying patients with chronic conditions using pharmacy data in Switzerland: an updated mapping approach to the classification of medications

**DOI:** 10.1186/1471-2458-13-1030

**Published:** 2013-10-30

**Authors:** Carola A Huber, Thomas D Szucs, Roland Rapold, Oliver Reich

**Affiliations:** 1 Department of Health Sciences, Helsana Insurance Group, P.O. Box, 8081 Zürich, Switzerland; 2 Institute of Pharmaceutical Medicine/European Center of Pharmaceutical Medicine, University of Basel, 4056 Basel, Switzerland

**Keywords:** Population health, Pharmacy data, Medication classification, Chronic conditions

## Abstract

**Background:**

Quantifying population health is important for public health policy. Since national disease registers recording clinical diagnoses are often not available, pharmacy data were frequently used to identify chronic conditions (CCs) in populations. However, most approaches mapping prescribed drugs to CCs are outdated and unambiguous. The aim of this study was to provide an improved and updated mapping approach to the classification of medications. Furthermore, we aimed to give an overview of the proportions of patients with CCs in Switzerland using this new mapping approach.

**Methods:**

The database included medical and pharmacy claims data (2011) from patients aged 18 years or older. Based on prescription drug data and using the Anatomical Therapeutic Chemical (ATC) classification system, patients with CCs were identified by a medical expert review. Proportions of patients with CCs were calculated by sex and age groups. We constructed multiple logistic regression models to assess the association between patient characteristics and having a CC, as well as between risk factors (diabetes, hyperlipidemia) for cardiovascular diseases (CVD) and CVD as one of the most prevalent CCs.

**Results:**

A total of 22 CCs were identified. In 2011, 62% of the 932′612 subjects enrolled have been prescribed a drug for the treatment of at least one CC. Rheumatologic conditions, CVD and pain were the most frequent CCs. 29% of the persons had CVD, 10% both CVD and hyperlipidemia, 4% CVD and diabetes, and 2% suffered from all of the three conditions. The regression model showed that diabetes and hyperlipidemia were strongly associated with CVD.

**Conclusions:**

Using pharmacy claims data, we developed an updated and improved approach for a feasible and efficient measure of patients’ chronic disease status. Pharmacy drug data may be a valuable source for measuring population’s burden of disease, when clinical data are missing. This approach may contribute to health policy debates about health services sources and risk adjustment modelling.

## Background

The evaluation of the population health status, patients’ health care needs and its associated costs is a priority issue in health policy, decision-making and resource allocation debates. In general, data of national disease registries and prevalence surveys, including clinical diagnoses according to the International Classification of Diseases (ICD-10-codes) have regularly been used to estimate the health status of a population. However, this type of data pool is not available in all health care systems. In Switzerland, for example, epidemiological data, providing information on the prevalence of chronic diseases and comorbidities in general population, are not widely available.

Administrative databases such as drug prescription data have thus been frequently used to identify persons with chronic conditions, quasi as an indirect method to estimate prevalence. Pharmacy based claims data provide a consistently available information source, which is reliable, covers a large population and might be extremely useful for assessment of morbidity [1-6]. Pharmacy-based diagnosis were used in risk adjustment models [7-9], illness severity measurement [10,11], prevalence estimates [12-15] and epidemiological studies for comorbidity adjustments [16,17]. However, in these studies the clustering of the Anatomical Therapeutic Chemical (ATC)-codes has not been applied consistently, and even in few studies the used ATCs are not documented. Moreover, the well-known ATC-algorithm of Lamers/van Vliet [18-20], the Pharmacy-based Cost Group (PCG) model, was often used in various modified and unspecified defined versions. The PCG model distinguishes 22 chronic conditions and was primarily developed to predict cost of diseases for risk adjustment. However, this model has some limitations. Medication classifications based on data, which have been recorded about 10 years ago. New drugs, which had not been developed and thus were not commercially available in the past years, were not included in the model. Furthermore, previous studies claimed the possibility of an exact differentiation between specific diseases via ATC code [12,19-21]. For example beta-blockers and diuretics were assigned to the category “hypertension” [19,22]. However, beta-blockers were also prescribed in patients with other cardiovascular diseases. Another example, diuretics were included in the category “cardiovascular diseases” although diuretics were also frequently used in patients with renal diseases [12,21]. In several medication classes, an ambiguous assignment of medication to chronic conditions is challenging and even, in certain cases, infeasible.

To overcome the limitations of previous mapping approaches, and to suggest a standardised and transparent use of the mapped medication classes to chronic conditions, we aimed to develop an updated mapping algorithm with a special focus on the unambiguous assignment of prescription drugs to chronic diseases. We provide an updated and rather conservative mapping approach to the classification of medications. Our classification is on the one side detailed as possible and on the other side we summarise categories to the superior category of disease when needed. Furthermore, we give an overview of the proportions of chronically ill patients in Switzerland using pharmacy data.

## Methods

### Study design and population

This study was a cross-sectional study covering all 26 cantons in Switzerland during the study period of January 1, 2011 to December 31, 2011. The study sample included all mandatory insured individuals aged 18 years or older insured by the Helsana Insurance Group, the leading Swiss health insurer. Individuals who died during the calendar year 2011 were also included in our study sample. In Switzerland, each resident has a mandatory basic coverage which is financed by a premium. All Swiss health insurants have an insurance coverage with cost sharing consisting of co-payments and deductibles. Co-payments are a charge of 10% of the health care costs per year which every person has to pay. This co-payment is limited to 700 Swiss francs (Sfr) per year. Deductibles range from 300 (Sfr) to a maximum of 2500 (Sfr) per year and can be chosen by the insurants. The amount of the premium decreases when the patient choose higher deductibles.

### Data source

Medical claims data were used from Helsana, covering about 1.3 million Swiss residents with mandatory health insurance. Population characteristics comprised gender, age, regional variables (e.g. language area) and the type of health insurance plan (managed care model, accident coverage, type of deductible class). The database also included information on health care visits, prescription drugs and drug costs. Drug data were based on medications prescribed in the outpatient setting including prescribed drugs which were purchased directly at the pharmacy. The outpatient setting comprised practice-based primary care physicians and specialists, as well as physicians from ambulatories, outpatient clinics and walk-in clinics. In our database, all prescribed drug items were coded according to the WHO ATC classification system [23]. Our (drug) data are highly reliable because the collected insurance claims covered almost all health care and pharmacy invoices. Since about 3% of the invoices were directly paid by the patient and not submitted for reimbursement, only a small percentage of invoices could not considered in our analyses. Permission to access the study data was provided by the Helsana Group.

### Identification of chronic diseases

The Anatomical Therapeutic Chemical (ATC) classification system was used to classify drugs and was controlled by the WHO Collaborating Centre for Drug Statistics Methodology. Each drug ingredient was assigned to the so called ATC code and was classified into different groups according to the human organ system on which they act. Ingredients can be used in different local application formulations: oral, inhalation, nasal, injection, installation, parenteral, rectal, transdermal, sublingual/buccal, implant, vaginal. Based on the WHO-report on ATC classification system, ATC-codes were assigned to chronic conditions by a medical expert group consisting of primary and secondary care physicians as well as health services researchers. We only included ATC-codes for chronic diseases which are exclusively used for the treatment of these diseases. Some drugs may be assigned to two chronic diseases simultaneously. For example, loop or “high-ceiling” diuretics (e.g. sulfonamides) are most frequently used in patients with heart failure but also in patients with poor renal function (chronic kidney disease). Additionally, beta-blockers are prescribed both in patients with hypertension and in patients with other cardiovascular diseases. In general, an unique assignment of ATC-codes to heart diseases is challenging. Since new trends in the use of various drugs for cardiac and hypertensive patients have been established over the last years, a clear distinction between ATC-codes for cardiac diseases and for hypertension is infeasible. Therefore, we conservatively evaluated the ATCs related to chronic diseases and generated relatively broad ATC-groups (e.g. “cardiovascular diseases inclusive hypertension”). ATCs, which could not be matched to a certain disease, were excluded. In our database, all ATC-codes referring to these ATC-groups classes were coded with the correspondingly chronic condition.

### Statistical analyses

We calculated the point prevalence of chronic conditions (CC) by dividing the number of patients with at least one prescribed drug in one of the generated ATC-groups CC at the end of the given year (31.12.2011) by the total number of insured persons aged ≥ 18 years at the end of 2011 (31.12). We presented proportions of patients with chronic conditions stratified by sex and age.

In this study, we also described the situation of patients with cardiovascular diseases. Cardiovascular diseases are the leading cause of death and are becoming an increasing economic and social burden for industrialized societies [24]. Therefore, we calculated prevalence rates of cardiovascular diseases (including hypertension) in combination with their risk factors, diabetes and hyperlipidemia, by age.

Furthermore, we estimated multiple logistic regression models to determine independent associations between age, sex, regional variables, the type of health insurance plan, and the occurrence of any chronic condition. Additionally, we performed a logistic regression to determine factors associated with the presence of cardiovascular diseases (including hypertension) as dependent variable. The independent variables included cardiovascular risk factors such as diabetes and hyperlipidemia, as well as patient characteristics described in the previous model. Analyses were performed using the software package R (version 2.12.2).

## Results

Based on prescription drug data, we identified a total of 22 diseases. Table 1 summarises the chronic conditions with the assigned medication classes.

**Table 1:** Chronic diseases and assigned ATC-codes and medication classes

**Chronic condition**	**ATC classification**	**Medication class**
Acid related disorders	A02	Antacids
		Drugs for peptic ulcer and gastroesophageal reflux disease (GERD)
		Other drugs for acid related disorders
Bone diseases (osteoporosis)	M05	Drugs for treatment of bone diseases
Cancer	L01	Antineoplastic agents
Cardiovascular diseases (incl. hypertension)	B01AA, B01AC,	Cardiac agents (excl. ACE inhibitors)
	C01, C04A,	Antihypertensives
	C02, C07,	Peripheral vasodilators
	C08, C09	Beta blocking agents
		Calcium channel blockers
		Agents acting on the renin-angiotensin system
		Vitamin K antagonists
		Platelet aggregation inhibitors (excl. herparin)
Dementia	N06D	Anti-dementia drugs
Diabetes mellitus	A10A, A10B,	Insulins and analogues
	A10X	Blood glucose lowering drugs (excl. insulins)
		Other drugs used in diabetes
Epilepsy	N03	Antiepileptics
Glaucoma	S01E	Antiglaucoma preparations and miotics
Gout, Hyperuricemia	M04	Antigout preparations
HIV	J05AE, J05AG,	Protease inhibitors
	J05AR	Non-nucleoside reverse transcriptase inhibitorsAntivirals for treatment of HIV infections, combinations
Hyperlipidemia	C10	Lipid modifying agents
Intestinal inflammatory diseases	A07EA, A07EC	Corticosteroids acting locallyAminosalicylic acid and similar agents
Iron deficiency anemia	B03AA, B03AB,	Iron bivalent, oral prepartations
		Iron trivalent, oral preparations
	B03AC	Iron trivalent, parenteral preparations
Migraines	N02C	Antimigraine preparations
Pain	N02A, N02B	Opioids
		Other analgesics
Parkinson’s disease	N04	Anti-parkinson drugs
Psycholgical disorders (sleep disorder, depression)	N05B, N05C,	Anxiolytics
	N06A	Hypnotics and sedativesAntidepressants
Psychoses	N05A	Antipsychotics
Respiratory illness (asthma, COPD)	R03	Drugs for obstructive airway diseases
Rheumatologic conditions	M01, M02	Antiflammatory and antirheumatic products
	L04AA, L04AB	Topical products for joint and muscular pain
		Selective immunosuppressants
		TNF-alpha inhibitors
Thyroid disorders	H03	Drugs for thyroid therapy
Tuberculosis	J04A	Drugs for treatment of tuberculosis

Table 2 shows the population characteristics of our study sample. We identified 932′612 adult individuals of a total of more than 1′300′000 subjects in 2011. There were slightly more woman (53%) than men. The mean age of the population was 51 years. About 40% of the sample chose a health insurance plan with managed care and 30% a deductible class over 500 Swiss francs. Approximately two thirds of the sample had one or more medication classes assigned with chronic conditions. Since several patients have been prescribed more than one disease related medication, multimorbidity could be observed. About 45% of the population had at least two chronic conditions (result not shown).

**Table 2:** Population characteristics of insured individuals in 2011

**Variables**	**N (%)**
Total	932′612
Gender	
Women	489′501 (52.5)
Mean age (sd*)	51.4 (19.2)
Language area	
German	715′443 (76.7)
French	151′144 (16.2)
Italian	64′040 (6.9)
Rhaeto-Romanic	1′985 (0.2)
Insurance plan	
Managed care	
Yes	394′362 (42.3)
No	538′250 (57.7)
Deductible class (> 500 Swiss francs/year)	
Yes	283′441 (30.4)
No	649′171 (69.6)
Accident coverage	
Yes	514′930 (55.2)
No	417′682 (44.8)
One or more chronic condition	
Yes	578′115 (62.0)
No	354′497 (38.0)

*Standard deviation.

**Figure 1:**
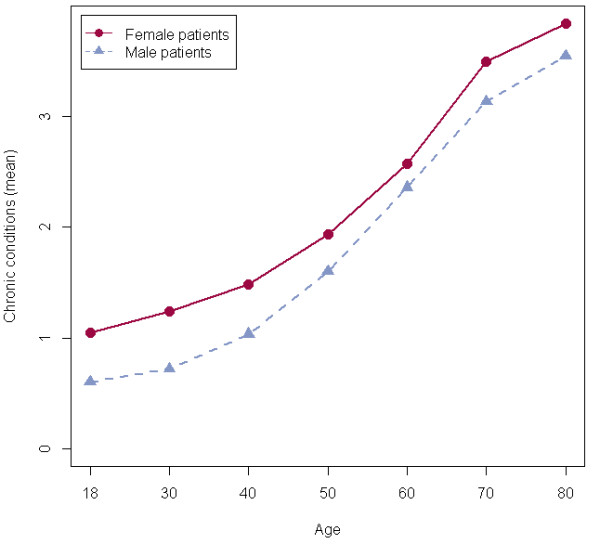
Mean number of chronic conditions among all patients by sex and age.

Within both gender groups, the number of medication classes assigned to chronic conditions increased linearly with age, as plotted in Figure 1. The strongest increase from approximately 1.5 to 3 chronic conditions was observed in patients aged between 40 and 70 years. The curve was moderately rising in younger (18–40 years) as well as in older age (>70 years). Female patients were prescribed slightly more drugs for chronic diseases compared to male patients. On average, patients suffered from about two chronic conditions in the age group of 50–59 years, from about three chronic conditions in the age group of 60–69 years and from slightly more than three conditions in the age group of over 69 years or older.

The number and proportions of insured persons in each ATC-group are presented in Table 3. The largest proportions of persons suffered from rheumatologic conditions (36%), cardiovascular diseases including hypertension (29%), pain (28%) and psychological disorders including sleep disorders and depressions (21%). About 20% of the individuals have been prescribed a drug for treating acid related disorders, 12% for hyperlipidaemia and 7% for respiratory illnesses including asthma and COPD.

**Table 3:** Number and proportion of patients by chronic condition

**Chronic condition**	**Number of patients**	**Proportion of patients (%)**
Acid related disorders	183′211	19.6
Bone diseases (osteoporosis)	22′159	23.8
Cancer	13′105	1.4
Cardiovascular diseases (incl. hypertension)	269′993	29.0
Dementia	16′780	1.8
Diabetes mellitus	50′751	5.4
Epilepsy	27′225	2.9
Glaucoma	33′640	3.6
Gout, Hyperuricemia	16′332	1.8
HIV	1′956	0.2
Hyperlipidemia	114′698	12.3
Intestinal inflammatory diseases	4′542	0.5
Iron deficiency anemia	38′022	4.1
Migraines	11′325	1.2
Pain	261′225	28.0
Parkinson’s disease	10′942	1.2
Psycholgical disorders (sleep disorder, depression)	197′490	21.2
Psychoses	30′024	3.2
Respiratory illness (asthma, COPD)	68′595	7.4
Rheumatologic conditions	336′373	36.1
Thyroid disorders	35′053	3.8
Tuberculosis	799	0.1

**Figure 2:**
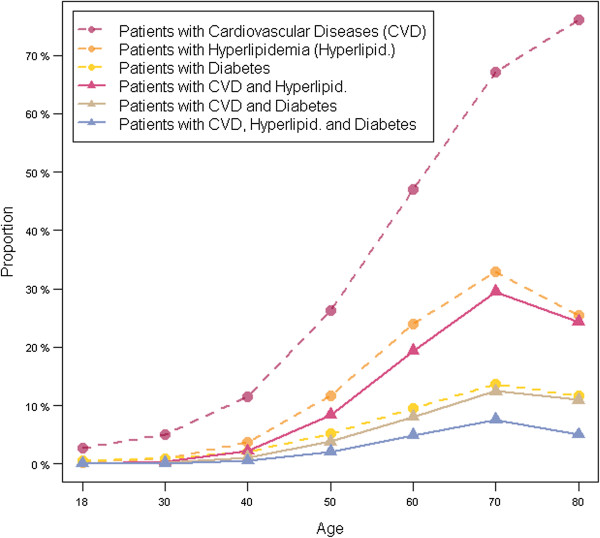
Proportions of patients with cardiovascular diseases and their risk factors by age.

29% of the persons had CVD, 10% both CVD and hyperlipidemia, 4% CVD and diabetes, and 2% suffered from all of the three conditions (results not shown). As showed in Figure 2, the prevalence of cardiovascular diseases (including hypertension), hyperlipidemia and diabetes was continuously increasing with age. Furthermore, there was a strong increase in the proportion of patients between 40 and 70 years suffering from cardiovascular diseases in combination with hyperlipidemia and/or diabetes mellitus. For the age group 40–49 years, the prevalence was 2.2% for cardiovascular diseases combined with hyperlipidemia, 1.1% for cardiovascular diseases with diabetes and 0.5% for cardiovascular diseases with hyperlipidemia and diabetes. For the age group of 70–79 years, the proportion of patients with cardiovascular diseases combined with hyperlipidemia was 29.5%, with diabetes 12.4% and with both hyperlipidemia and diabetes 7.5%. With exception of cardiovascular diseases, every proportion of patients with chronic condition and its combinations slightly decreased in older age groups (70–79 and >79 years). Additional analyses could show that 35% of patients with cardiovascular diseases were also treated for hyperlipidemia and 15% for diabetes mellitus. About 9% of the patients with cardiovascular diseases were both hypertensive and diabetic (results not shown).

To identify independent predictors of the occurrence of any chronic condition, we estimated a multiple logistic regression model (Table 4). Female sex and older age strongly predicted the prevalence of any chronic condition. We constructed a second multiple logistic regression to determine risk factors for prevalent cardiovascular diseases. Older age and suffering from diabetes mellitus and hyperlipidemia were independently associated with prevalent cardiovascular diseases (inclusive hypertension).

**Table 4:** Risk factors for chronic conditions in multivariate modelling using logistic regression

	**Chronic condition**		**Cardiovascular diseases**	
**Variable**	OR*	**95%-CI**^**†**^	OR*	**95%-CI**^**†**^
Gender				
Women	1.39	1.38-1.41	0.89	0.88-0.90
Age groups				
18-29	1.00		1.00	
30-39	1.50	1.48-1.53	2.16	2.07-2.24
40-49	1.65	1.62-1.67	4.20	4.05-4.36
50-59	2.27	2.23-2.31	8.85	8.55-9.16
60-69	3.95	3.89-4.02	17.20	16.62-17.79
70-79	7.79	7.62-7.97	34.44	33.24-35.68
>79	8.73	8.50-8.97	60.80	58.61-63.07
Language area				
German	1.00		1.00	
French	1.25	1.23-1.26	0.87	0.85-0.88
Italian	1.11	1.09-1.14	0.84	0.82-0.86
Rhaeto-Romanic	0.82	0.74-0.90	0.77	0.67-0.88
Insurance plan				
Managed care	1.001	0.99-1.01	0.92	0.91-0.93
Deductible class (> 500 Swiss francs/year)	0.25	0.25-0.25	0.36	0.35-0.36
Accident coverage	1.06	1.05-1.07	1.15	1.13-1.16
Cardiovascular risk factors				
Diabetes			4.23	4.11-4.34
Hyperlipidemia			6.79	6.67-6.92
Nagelkerke R2	0.28		0.52	

*Odds ratio.

†95% confidence interval.

Table 5 gives an overview on our estimated proportions of patients with cardiovascular diseases, diabetes mellitus, hyperlipidemia, cancer and respiratory illness (asthma, COPD) compared to those proportions from available data sources in Switzerland [25-35]. Proportions of cancer, cardiovascular diseases, diabetes and respiratory illness reported in previous studies were similar to our estimates using pharmacy data [25,28,31,34]. However, previous findings about the prevalence of hyperlipidemia were about twice as high than those estimated in our pharmacy based study [27,33].

**Table 5:** Proportions of chronically ill patients compared to other epidemiological data

**Chronic condition**	**Various data sources**				**Data used in this study (2011)**
	**Proportion of patients (%)**	**Description of the chronic condition**	**Data source/study design (year of data)**	**Reference**	**Proportion of patients (%)**
Cancer	1.5	All malignant neoplasms excluding non-melanoma skin cancer	Registry data (1992)	Micheli et al. 2002 [25]	1.4
	1.4		Registry data (2008)	WHO 2008 [26]	
Cardiovascular diseases (incl. hypertension)	30.0	Hypertension	Epidemiological data (2001)	Pechère-Bertschi et al. 2005 [27]	29.0
	36.0	High blood pressure	Epidemiological data (2003–2006)	Firmann et al. 2008 [28]	
	34.4	Hypertension	Epidemiological data (1999-2009)	Guessous et al. 2012 [29]	
Diabetes mellitus	6.3	Type 2 diabetes	Epidemiological data (2003–2006)	Kaiser et al. 2012 [30]	5.4
	5.8	Diabetes	Officially registered hospital discharges with diagnosis (2008)	Bopp et al. 2011 [31]	
	3.3	Non-insulin dependent diabetes	Electronic medical records (2009–2011)	Rizza et al. 2012 [32]	
Hyperlipidemia	20.0	Dyslipidemia	Epidemiological data (2001)	Pechère-Bertschi et al. 2005 [27]	12.3
	29.0	Dyslipidemia	Epidemiological data (2003–2006)	Firmann et al. 2010 [33]	
Respiratory illness (asthma, COPD)	5.7-10.0	Asthma	Epidemiological data (2002)	Bridevaux et al. 2010 [34]	7.4
	9.1	COPD	Epidemiological data (2002)	Bridevaux et al. 2008 [35]	

Proportions of patients with cancer, respiratory illness, cardiovascular diseases and cardiovascular risk factors based on data used in this study (2011) compared to proportions from available data sources in Switzerland.

## Discussion

This study developed an updated mapping approach to identify chronically ill patients using pharmacy data. Databases on prescribed drugs are an useful source of information on morbidity, if reliable medical diagnoses are missing.

In our classification model, we identified a total of 22 chronic conditions based on the 2011 WHO-ATC-classification. We found that the largest proportions of persons suffered from pain, rheumatologic conditions and cardiovascular diseases, including hypertension. These findings are in line with previous studies in Italy estimating prevalence of chronic diseases showing highest rates in cardiovascular diseases and rheumatologic conditions [12,13]. On the other hand, our results are different from the Dutch estimates based on the PCG-model showing a low prevalence rate in rheumatologic conditions [19]. One explanation is the fact that a different and partly out-dated medication classification for the chronic conditions was used. New drugs, which have been developed during the last decade, were not included in the PCG-model performed in the nineties. We assume that the Italian results may be more exact, since a more recent medication classification was used in comparison to the Dutch study. Several of our estimated proportions of patients with chronic conditions are comparable to prevalence estimates in other European countries. For example, the prevalence of diabetes mellitus, as one of the leading health problems in the industrialised countries, is similar to the prevalence in Germany and the Netherlands [15,36,37]. Additionally, the prevalence rates of asthma are comparable to those reported in other European countries such as France, Norway and Germany [38]. Not surprisingly and in line with several studies, we could show that chronic conditions affected all ages, but with a significant increase in older age groups [39-42]. Furthermore, we found that cardiovascular diseases are mainly attributable to classical risk factors such as diabetes and hyperlipidemia. A large body of epidemiological and pathological data also demonstrated that diabetes and dyslipidemia are independent risk factors for cardiovascular diseases in both men and women. Diabetes and hyperlipidemia are common comorbidities of cardiovascular diseases and contributed significantly to its overall mortality [43-47].

A further aim of our study was to compare our estimated proportions of chronically ill patients with the reliable prevalence estimates from national studies. Therefore, we compared our estimated proportions of patients with cardiovascular diseases, diabetes mellitus, hyperlipidemia, cancer and respiratory illness (asthma, COPD) to those proportions from available data sources in Switzerland. Data on further chronic conditions could not be presented, since epidemiological data in Switzerland are very scarce. We found a high level of agreement between the estimates using pharmacy data and those reported by epidemiological studies. Proportions of cancer, cardiovascular diseases, diabetes and respiratory illness matched quite well [25,28,31,34]. For hyperlipidemia, study results showed lower prevalence estimates than those revealed in our pharmacy based study [27,33]. One explanation for the discrepancy between our findings and those from Pèchere-Bertschi et al. [27] and Firmann et al. [33] is the questionable representativeness for the general Swiss population. For example, Firmann et al. [33] collected data from a sample of 6′084 subjects living in one Swiss city, Lausanne, with a relatively high percentage of non-Swiss compared to other cities. Moreover, this study was conducted six years ago and recent trends in prevalence are not taken into account.

There are some limitations addressing the accuracy of our estimates. About 3% of the used claims invoices were paid directly by the patients and not by the health insurer. Drug data may be underrepresented since claims data are not completely recorded. Furthermore, the number of prescribed drugs was underreported because they were obtained exclusively in outpatient settings. Additionally, drug-based diagnoses are naturally regarded as a proxy for a medical diagnosis. Since we excluded ATC-codes which could not be matched to certain diseases, proportions may be underestimated. Moreover, we are not able to distinguish specific chronic diseases such as hypertension from other cardiovascular diseases. For legal and ethical reasons as well as regarding data security, data do not include medical diagnoses and clinical procedures, and thus we are unable to give more information on chronic conditions as well as to provide prevalence estimates based on medical diagnoses (e.g. ICD-10) for comparisons in the same data source. Furthermore, in Switzerland reliable epidemiological data are scare. Appropriate additional, independent samples for comparing the prevalence estimates are not widely available. However, drug-based diagnoses could not completely substitute, but add clinical data. Mapping medication classes to chronic conditions is a frequently recommended and used in previous studies [10,12,21]. Moreover, we assume that no patient is prescribed a drug for chronic conditions such as insulin without a serious indication of diabetes. However, this study has several strengths. Our study is based on a very comprehensive administrative claims database for a large population across Switzerland with an almost complete recording of prescribed drug data. Administrative data are a consistently available source of information on morbidity, health care use and costs of care. They are reliable, longitudinal, practice-based, large in size and widely accepted in epidemiological, health services and outcomes research to assess prevalence. Moreover, studies developing risk models emphasised that prescription data derived from administrative claims database have several advantages over diagnostic data regarding reliability, timeliness and completeness of data [7,11,48]. In the Dutch health care system, the use of information on chronic conditions obtained from claims for prescribed drugs are well established as a helpful instrument for improving risk equalisation between the sickness funds as well as in capitation modelling [19,20,49]. In Switzerland, there is an on-going debate about the optimal risk equalisation scheme [50-53]. The present Swiss risk equalisation scheme is a demographics-based risk equalisation model which based on age, sex and an at least three successive night-stays in a hospital or nursing home (in the previous year) as equalising elements. In 2011, the Swiss parliament has decided to refine the risk equalisation by diagnoses based on pharmacy data, since no appropriate data on diagnosed diseases exist. Consequently, it is particularly important to identify those ATC-codes which are exclusively used for the treatment of population’s common diseases. Pharmacy-based, estimated prevalence data might be a useful addition for risk adjustment modelling in health care delivery systems with demographics-based risk equalisation schemes.

## Conclusions

Using pharmacy data, we developed an updated and improved approach for a feasible and efficient measure of patients’ chronic disease status. Pharmacy drug data may be a valuable source for measuring population’s burden of disease, when clinical data are missing. This approach may contribute to health policy debates about health services sources and risk adjustment modelling.

## Competing interests

All authors declare that they have no competing interests.

## Authors’ contributions

CAH and OR participated in the conceptual development and study design. CH drafted the manuscript and RR analysed the data. OR and TDS revised the manuscript. All authors participate in the interpretation of data, critically reviewed and gave final approval to the manuscript.

## Pre-publication history

The pre-publication history for this paper can be accessed here:

http://www.biomedcentral.com/1471-2458/13/1030/prepub
